# The Role of Inflammasome in Abdominal Aortic Aneurysm and Its Potential Drugs

**DOI:** 10.3390/ijms25095001

**Published:** 2024-05-03

**Authors:** Suyu Pi, Sizheng Xiong, Yan Yuan, Hongping Deng

**Affiliations:** 1 Department of Vascular Surgery, Renmin Hospital of Wuhan University, Wuhan 430060, China; 15072198615@163.com (S.P.); rmxiongsz@whu.edu.cn (S.X.); dryuan1218@whu.edu.cn (Y.Y.); 2 Aortic Abdominal Aneurysm (AAA) Translational Medicine Research Center of Hubei Province, Wuhan 430060, China

**Keywords:** abdominal aortic aneurysm, inflammasome, NLRP3, AIM2, inflammation, activation and regulation, potential drugs

## Abstract

Abdominal aortic aneurysm (AAA) has been recognized as a serious chronic inflammatory degenerative aortic disease in recent years. At present, there is no other effective intervention except surgical treatment for AAA. With the aging of the human population, its incidence is increasing year by year, posing a serious threat to human health. Modern studies suggest that vascular chronic inflammatory response is the core process in AAA occurrence and development. Inflammasome, a multiprotein complex located in the cytoplasm, mediates the expression of various inflammatory cytokines like interleukin (IL)-1β and IL-18, and thus plays a pivotal role in inflammation regulation. Therefore, inflammasome may exert a crucial influence on the progression of AAA. This article reviews some mechanism studies to investigate the role of inflammasome in AAA and then summarizes several potential drugs targeting inflammasome for the treatment of AAA, aiming to provide new ideas for the clinical prevention and treatment of AAA beyond surgical methods.

## 1. Introduction

Abdominal aortic aneurysm (AAA) has been recognized as a serious chronic inflammatory degenerative aortic disease in recent years, and it is characterized by the progressive pathological dilatation of the abdominal aortic wall [1,2]. AAA can be diagnosed when the diameter of a permanently dilated or bulging abdominal aorta is greater than 50% of normal or the diameter of the abdominal aorta is ≥3 cm in general [3]. Most patients who develop AAA are usually asymptomatic; however, when the aneurysm expands and ruptures, its mortality is extremely high [4]. According to reports, even if ruptured AAAs are treated in time, the cases fatality rate is still as high as 50–70%, coupled with the cases without timely surgery, the ruptured AAAs’ total mortality can be as high as 90% [5]. Its risk factors include smoking, gender, age, hypertension, chronic obstructive pulmonary disease, hyperlipidemia, and family history of AAA [6,7,8]. Among all ethnic groups, African Americans, Asians, and Hispanics are at lower risk than Caucasians [9]. Diabetes mellitus is a common comorbidity of atherosclerotic disease, although the incidence of AAA in diabetic patients is low [10].

Current guidelines recommend surgical treatment for AAA if the maximum diameter is at least 5.5 cm in men (or 5.0 cm in women). Surgery is also recommended for rapidly progressive AAA (≥1 cm/year), symptomatic AAA, ruptured AAA, and AAA with eccentric or cystic structures [11,12]. Surgery is divided into open surgery and endovascular repair surgery, but both have corresponding defects and complications. For example, open surgery for the treatment of abdominal aortic aneurysm is technically mature and has good long-term effects, but its main drawbacks are significant surgical trauma, a large amount of bleeding, and long postoperative recovery time [13]. Although endovascular repair surgery is minimally invasive, it can be interfered with by the anatomical conditions of the aneurysm, which may lead to a severe insufficient anchoring area, resulting in serious complications such as internal leakage or stent displacement, so the widespread use of endovascular techniques has also been limited [14,15]. In addition, the management of small AAA (diameter less than 5 cm) is mainly focused on monitoring, and there is no clear drug that can alleviate the progression of small AAA. Clinical trial results show that statins or doxycycline have no obvious inhibitory effect on the growth of AAA [16]. As a result, it is crucial to clarify the molecular regulatory mechanisms of AAA occurrence and development in order to identify potential drug targets.

Modern studies have identified aortic extracellular matrix (ECM) degradation, the apoptosis of vascular smooth muscle cells (VSMCs), and vascular chronic inflammatory response as the three basic pathological processes in the pathogenesis of AAA [17]. Of these, vascular chronic inflammatory response is the core process. The cytokines released by inflammatory cells not only exacerbate ECM degradation but also lead to the apoptosis of VSMCs [18]. For example, interleukin (IL)-1β, IL-6, IL-33, and other stimuli prompt macrophages or VSMCs to secrete matrix metalloproteinases (MMPs) that degrade elastin and collagen, leading to the apoptosis of VSMCs and ECM degradation, thereby disrupting the stability of the aortic wall architecture [19]. It has been demonstrated in animal experiments that the use of an IL-1β receptor inhibitor (anakinra) can effectively inhibit mouse AAA formation induced by porcine pancreatic elastase (PPE) perfusion [20]. Therefore, inflammasome regulating the secretion of cytokines like IL-1β and IL-18 may significantly influence AAA progression, which has been recognized as a chronic inflammatory disease.

## 2. Inflammasome

As a significant component of the innate immune system, inflammasome has been one of the research hotspots in the field of innate immunity since it was first proposed by Martinon et al. [21] in 2002. Inflammasome, a multiprotein complex located in the cytoplasm, is mainly expressed in monocytes/macrophages and can also be expressed in dendritic cells, neutrophils, and certain non-immune cells [22,23]. At present, the inflammasomes that have been found mainly include NLRP1 inflammasome, NLRP3 inflammasome, NLRC4 inflammasome, IPAF inflammasome, and AIM2 inflammasome. Inflammasome mainly consists of three components: receptor protein (a member of the NLR or ALR family), adaptor protein apoptosis-associated speck-like protein containing a caspase recruitment domain (ASC), and effector protein caspase-1 [24,25].

Therefore, as a multiprotein complex assembled by pattern recognition receptors (PRRs) in the cytoplasm, inflammasome can recognize and amplify various injury or stimulation signals from pathogen-associated molecular patterns (PAMPs) or damage/danger-associated molecular patterns (DAMPs), thereby playing a central role in mediating inflammation [26,27]. The activation of inflammasome promotes the self-cleavage of pro-caspase-1 to produce the active effector protein caspase-1, which further cleaves pro-IL-1β, pro-IL-18, and gasdermin D (GSDMD). Immediately, the N-terminal fragment of GSDMD (GSDMD-N) forms oligomeric pores in the cell membrane that mediate the osmotic swelling and death of inflammatory cells (e.g., macrophages), followed by releasing inflammatory factors like IL-1β and IL-18, which was formerly known as the pyroptosis of inflammatory necrosis [28]. Apart from this canonical pathway, pyroptosis and inflammasome activation can also be mediated through the non-canonical caspase-4/5/11 pathway or a newly discovered caspase-3/gasdermin E (GSDME) pathway [29,30]. Thus, pyroptosis has been redefined as gasdermin-mediated lytic cell death [31].

Recent research has focused more on NLRP3 inflammasome and AIM2 inflammasome, which have been shown to play important roles in various macrophage-driven inflammation-related diseases. Moreover, studies have continuously pointed out that inflammasome activation is a notable link in the pathogenesis of aneurysms. For example, in human cerebral aneurysm tissue, the expression levels of NLRP3 inflammasome components and their downstream products were as follows: ruptured group > unruptured group > normal group [32]. Moreover, the genetic deletion of NLRP3 or pharmacological inhibition of NLRP3 inflammasome with glyburide reduced the rate of thoracic aortic aneurysm formation in mice induced by high-fat diet and angiotensin II (Ang II) [33]. Thus, the following sections provide an in-depth analysis of the mechanisms of these two inflammasomes in AAA and discusses potential targeted drugs.

## 3. NLRP3 Inflammasome in AAA

**Figure 1:**
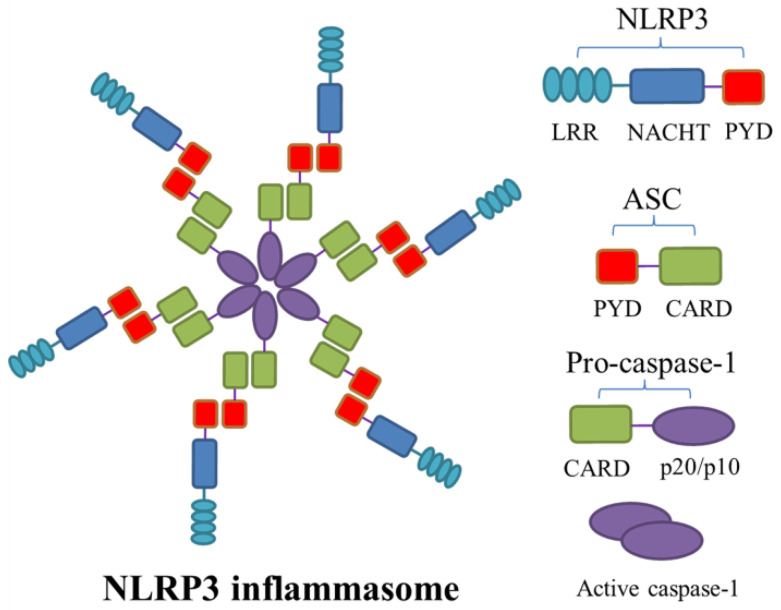
The structure of NLRP3 inflammasome. Nucleotide-binding oligomerization domain-like receptor family pyrin domain-containing 3 (NLRP3) inflammasome is a multiprotein complex assembled using three components: the adaptor apoptosis-associated speck-like protein containing a caspase-recruitment domain (ASC), NLRP3, and pro-caspase-1. When the central protein NLRP3 is stimulated by activation signals, ASC interacts with the N-terminal pyrin domain (PYD) of NLRP3 via homotypic PYD, followed by recruiting pro-caspase-1 via the C-terminal caspase recruitment domain (CARD)–CARD interactions, resulting in the oligomerization of inflammasome. Pro-caspase-1 subsequently cleaves itself into caspase-1 and then releases catalytically active subunits p20/10. LRR, leucine-rich repeat; NACHT, nucleotide-binding oligomerization domain.

Nucleotide-binding oligomerization domain-like receptor family pyrin domain-containing 3 (NLRP3) inflammasome is one of the well-characterized inflammasomes discovered in recent years, with a relative molecular mass of approximately 700 kDa. NLRP3 inflammasome is composed of its central protein NLRP3, adaptor protein ASC, and effector protein caspase-1. The central protein NLRP3 also includes the following three parts: the C-terminal leucine-rich repeat domain (LRR), which is responsible for ligand recognition; the nucleotide-binding oligomerization domain (NOD/NACHT) in the middle, which is the core structure of inflammasome and exhibits ATPase activity and mediates oligomerization of NLRP3 molecules; and the N-terminal pyrin domain (PYD), involved in signal transduction [34]. As an intracellular adaptor protein, ASC consists of two parts, the N-terminal PYD and the C-terminal caspase recruitment domain (CARD). When the central protein NLRP3 is stimulated by activation signals, ASC interacts with the PYD of NLRP3 via homotypic PYD, followed by recruiting pro-caspase-1 with CARD–CARD interactions, resulting in inflammasome oligomerization (Figure 1) [35]. Then, pro-caspase-1 cleaves itself into active caspase-1, which subsequently mediates pro-IL-1β, pro-IL-18, and GSDMD to cleave into active subunits, triggering pyroptosis and inflammation [36]. Due to its ability to be activated by manifold types of pathogens or danger signals, NLRP3 inflammasome can play a pivotal role in various disease processes, namely, cryopyrin-associated periodic syndromes (CAPS) [37,38], Alzheimer’s disease [39], rheumatoid arthritis [40], and others [41]. Additionally, many studies have suggested a correlation between NLRP3 inflammasome and AAA in recent years.

For example, in 2011, Roberts et al. [42] used a comparative detection of arterial wall samples from 1151 AAA patients and 727 non-AAA patients to establish that NLRP3 homozygous gene rs35829419 and its downstream product IL-1β were significantly overexpressed in the AAA group, suggesting that NLRP3 inflammasome did have a direct or indirect regulatory effect on AAA occurrence. In a subsequent study, NLRP3 and caspase-1 were found to be up-regulated in macrophages from human and mouse AAA tissues, while knockout of NLRP3 and caspase-1 revealed that the incidence and severity of AAA in mice induced by Ang II, the infiltration of inflammatory cells, and the expression of mitochondrial reactive oxygen species (mtROS) in adventitial macrophages were all inhibited [43]. The above outcomes suggested that Ang II might promote the assembly of NLRP3 inflammasome in macrophages and IL-1β release through angiotensin II type 1 (AT1) receptor or the mtROS-dependent pathway, leading to the generation of inflammation in the early stage of AAA [43]. Wu et al. [44] also reported an interesting finding in 2017, which demonstrated that caspase-1 could directly cleave and degrade contractile proteins of VSMCs at the arterial wall, showing that NLRP3 inflammasome might play a novel role in AAA. In addition, Ren et al. [45] observed that NLRP3 inflammasome in macrophages directly activated MMP-9 by cutting its own N-terminal inhibitory domain, while using a strong selective NLRP3 inflammasome inhibitor MCC950 to block inflammasome activation in macrophages could prevent the formation of an aortic aneurysm. Likewise, hyperhomocysteinemia (HHcy) exacerbated two different murine models of AAA by inducing the NLRP3 inflammasome activation in macrophages to stimulate inflammation, whereas the administration of folic acid reversed the HHcy-aggravated AAA with attenuated outer membrane inflammasome activation [46]. In short, the silence of NLRP3 inflammasome significantly diminishes AAA formation.

The results of Sun et al.’s [28] recent study indicated that the P2X7 receptor was highly expressed in calcium chloride (CaCl_2_) and Ang II-induced mouse AAA models, as well as in human AAA specimens, and co-localized with the macrophage marker CD68. Mechanically, the macrophage-derived purinergic receptor P2X7 is a ligand-gated cation channel. When activated by extracellular ATP, the P2X7 receptor promotes the influx of Na^+^ and Ca^2+^, as well as the efflux of K^+^, subsequently mediates downstream NLRP3 inflammasome activation, and then initiates canonical pyroptosis. The ensuing vascular inflammatory response propels an increase in MMPs and ROS, thus facilitating the formation of AAA. Furthermore, either the knockout of the P2X7 receptor or the use of the P2X7 receptor antagonist A438079 notably reduced the formation of AAA in experimental mouse models, whereas the agonist ATP-γ-S remarkably accelerated AAA progression [28]. Previously, another study reported that Pannexin-1 (Panx1) on vascular endothelial cells, a channel that releases ATP, stimulated macrophage activation by activating P2X7 receptor and mitochondrial DNA (mtDNA) release to increase the secretion of IL-1β as well as high-mobility group protein B1 (HMGB1, a notable late-stage inflammatory mediator), followed by promoting the formation of AAA [47].

## 4. AIM2 Inflammasome in AAA

**Figure 2:**
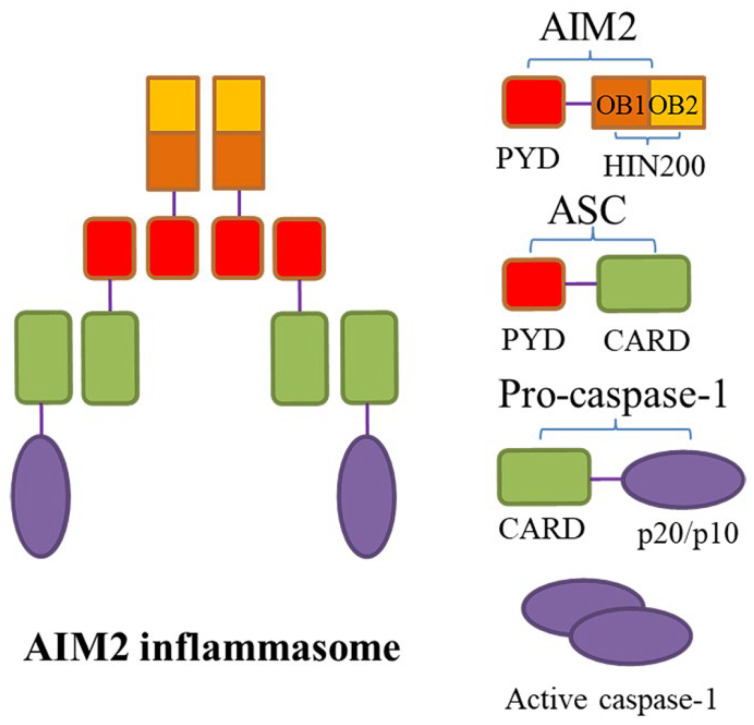
The structure of AIM2 inflammasome. Absent in melanoma 2 (AIM2) inflammasome is a macromolecular complex composed of AIM2, apoptosis-associated speck-like protein containing a caspase-recruitment domain (ASC), and caspase-1. AIM2 has two important domains, namely, the N-terminal pyrin domain (PYD) and the C-terminal hematopoietic interferon-inducible nuclear antigens with 200 amino acid repeats (HIN-200) domain. The latter contains two oligonucleotide/oligosaccharide-binding (OB) folded subdomains with high affinity for DNA. The PYD and HIN-200 domain interact to maintain AIM2 molecular self-inhibition during the steady state. When the HIN-200 domain is stimulated by the presence of cytoplasmic double-stranded DNA (dsDNA), self-inhibition of AIM2 is removed, ASC acts as an adaptor protein that connects upstream AIM2 with downstream pro-caspase-1 via the N-terminal PYD-PYD interactions and the C-terminal recruitment domain (CARD)–CARD interactions, leading to the assembly of AIM2 inflammasome; subsequently, pro-caspase-1 self-cleaves and its active subunits p20/10 are released.

Absent in melanoma 2 (AIM2) is a 344-amino acid protein with a relative molecular mass of about 39 kDa, encoded by the AIM2 gene (1485 kb) on human chromosome 1q22, which belongs to the interferon-inducible gene family [48]. AIM2 is the only known member of the natural immunoreceptor family that recognizes the presence of cytoplasmic double-stranded DNA (dsDNA) to assemble AIM2 inflammasome, which is a macromolecular complex including AIM2, adaptor protein ASC, and caspase-1 [49,50]. AIM2 can be expressed in vascular cells like macrophages, arterial endothelial cells, and VSMCs [51], localized in the cytoplasm, mitochondria, and nucleus [52]. It has two important domains, namely, the N-terminal PYD and the C-terminal hematopoietic interferon-inducible nuclear antigens with 200 amino acid repeats (HIN-200) domain. The latter has the advantage of dsDNA recognition [53], as it contains two oligonucleotide/oligosaccharide-binding (OB) folded subdomains with high affinity for DNA, which are 70 to 150 amino acids in length and used for binding to single-stranded DNA [54]. During the steady state, the PYD and HIN-200 domain interact to maintain AIM2 molecular self-inhibition, while the binding of the HIN-200 domain to the sugar phosphate backbone of dsDNA can relieve the self-inhibition of AIM2 [55]. Thus, in the AIM2 inflammasome, AIM2 acts as an initiator protein that specifically recognizes and binds cytosolic dsDNA through its HIN-200 domain, while ASC connects upstream AIM2 with downstream pro-caspase-1-activating components, thereby priming a canonical activation pathway similar to that of the NLRP3 inflammasome, which triggers pyroptosis and inflammatory response (Figure 2) [56]. Remarkably, it has also been found that AIM2 inflammasome could induce the non-canonical NLRP3 inflammasome activation via the pores formed by GSDMD and subsequent K^+^ efflux [57]. Over recent years, many studies have demonstrated the correlation of AIM2 inflammasome activation with AAA development as well.

In 2014, Dihlmann et al. [58] initially found that compared with a normal aorta, AAA specimens with higher inflammatory grade had higher expression levels of AIM2 inflammasome components. Moreover, compared to patients with lower risk of aneurysm rupture, the expression of AIM2 inflammasome core components (e.g., ASC, caspase-1, caspase-5) and its downstream product IL-1β were all conspicuously up-regulated in the peripheral blood single infiltrating lymphocytes of patients at higher risk, suggesting a correlation between AIM2 inflammasome and AAA rupture as well as indicating that AAA-associated lymphocytes could carry out AIM2 inflammasome signaling [58]. Another study [59] reported that the expression of AIM2 in peripheral blood leukocytes such as granulocytes, monocytes, B lymphocytes, and T lymphocytes was significantly increased in AAA patients, while the expression levels of other inflammasomes apparently did not differ between the two groups. After rendering exogenous DNA stimulation in vitro, IL-1β released by peripheral blood mononuclear cells (PBMCs) was remarkably higher than that of the control group, revealing that AIM2 inflammasome has a specific role in AAA development [59]. As indicated by a previous study [60], the expression levels of AIM2 inflammasome and downstream products in the PBMCs of AAA patients were obviously higher than those of non-AAA patients. However, all of these were reflected in male AAA patients, and there was no distinct difference between female patients and the control group. As a consequence, it was pointed out that male PBMCs display a systemic pro-inflammatory state, whereas primed inflammasomes might be partly responsible for the pathogenesis of AAA [60]. 

## 5. Inflammation Is a Common Feature in AAA and Atherosclerosis

AAA and atherosclerosis are two different but closely related diseases, and abdominal aortic atherosclerosis is common in middle-aged patients with AAA [61,62]. They share many similar features, such as inflammatory cell infiltration, matrix degradation, and arterial wall thrombosis, among which aseptic inflammation is a prominent common feature of these two diseases [63]. Therefore, the analysis of inflammation in atherosclerosis may help to deepen our understanding of the pathogenesis of AAA. Numerous previous studies have shown that inflammation in AAA and atherosclerosis is mainly mediated by the NLRP3 inflammasome [43,64]. In addition to classic cholesterol crystals in atherosclerotic plaques, which are one of the most potent DAMPs to activate NLRP3 inflammasome in macrophages [64,65], calcium phosphate crystals and crystals formed by saturated fatty acids also induce NLRP3 inflammasome activation [66,67]. Some reports have suggested that P2X7 receptor or hypoxia-mediated K^+^ efflux and mtROS/mtDNA production contribute to NLRP3 activation, and the subsequent release of active IL-1β induces vascular inflammation and ultimately leads to atherosclerosis [68,69,70]. Although both are chronic inflammatory diseases, AAA is not significantly associated with low-density lipoprotein, and there is no serious ECM degradation in atherosclerosis, hinting that the mechanisms of NLRP3 inflammasome activation are different between the two. Therefore, it is necessary to conduct the in-depth analysis and exploration of the activation and regulatory mechanisms of the inflammasome in such diseases, which is also the direction of subsequent research.

## 6. Activation and Regulation Mechanisms of the Inflammasome

**Figure 3:**
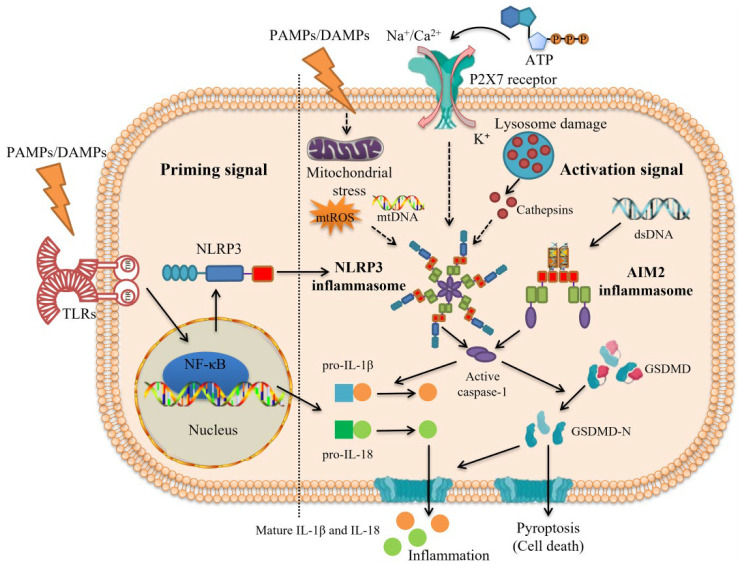
Two consecutive signals are requisite for inflammasome activation. The activation of inflammasome requires the priming signal (Signal 1) and the activation signal (Signal 2). Priming signal (Signal 1): pathogen-associated molecular patterns (PAMPs) or damage/danger-associated molecular patterns (DAMPs) such as lipopolysaccharides (LPS) act as priming signals to activate the nuclear factor kappa-light-chain-enhancer of activated B cells (NF-κB) signaling pathway through binding to Toll-like receptors (TLRs), thereby increasing the expression of downstream proteins such as nucleotide-binding oligomerization domain-like receptor family pyrin domain-containing 3 (NLRP3), pro-interleukin (IL)-1β, and pro-IL-18. Activation signal (Signal 2): under the stimulation of PAMPs/DAMPs, K^+^ efflux and the presence of cytoplasmic double-stranded DNA (dsDNA) are common activation signals that induce the assembly of NLRP3 inflammasome and absent in melanoma 2 (AIM2) inflammasome, respectively, and other upstream events including lysosome-released cathepsins, mitochondrial reactive oxygen species (mtROS), and mitochondrial DNA (mtDNA) are also needed for NLRP3 inflammasome activation. Then, inactive pro-caspase-1 transforms into active caspase-1 via self-cleavage, which subsequently mediates pro-IL-1β and pro-IL-18 to cleave into mature IL-1β and IL-18, triggering inflammation. Gasdermin D (GSDMD) is also cleaved by caspase-1 into the N-terminal fragment of GSDMD (GSDMD-N), which forms oligomeric pores in the cell membrane and releases inflammatory factors such as IL-1β and IL-18, leading to pyroptosis.

It is worth noting that recent studies have shown that the activation of inflammasome in macrophages needs two consecutive signals: the priming signal (Signal 1) and the activation signal (Signal 2) [71]. First, PAMPs/DAMPs act as priming signals to bind to Toll-like receptors (TLRs) and then activate the nuclear factor kappa-light-chain-enhancer of activated B cells (NF-κB) signaling pathway, thereby increasing the expression of downstream proteins (e.g., NLRP3, pro-IL-1β, and pro-IL-18) [72]. Then, K^+^ efflux and the presence of cytoplasmic dsDNA are activation signals that induce the assembly of NLRP3 inflammasome and AIM2 inflammasome, respectively. Furthermore, NLRP3 inflammasome activation is usually carried out with the help of lysosome-released cathepsins, mtROS, and mitochondrial DNA (mtDNA) (Figure 3) [73]. As Shridas et al. [74] found, the lack of serum amyloid A (SAA) could prevent the formation of AAA in mice induced by Ang II, whereas its increase contributed to NLRP3 inflammasome activation in macrophages and the increase in IL-1β secretion. Mechanistically, SAA may activate NLRP3 inflammasome in the form of ROS production, cathepsin B activation, and K^+^ efflux. Interestingly, high-density lipoprotein (HDL) eliminated the ability of SAA to stimulate ROS formation and inflammasome activation, which might be because, as a lipophilic apolipoprotein, many properties of SAA are lost when combined with HDL [75].

Post-translational modifications (PTMs), like phosphorylation and de-ubiquitination, have been revealed to induce the rapid priming of NLRP3 protein [63]. Similarly, other ion flux events (e.g., Ca^2+^ mobilization, Na^+^ influx, and Cl^−^ efflux) are also thought to promote the activation of NLRP3 inflammasome, but their definite regulatory mechanisms are not comprehended completely and still contentious [76]. Some recent studies have found that upon inflammasome being activated by Signal 2, NIMA-related kinase 7 (NEK7) can regulate NLRP3 inflammasome oligomerization and activation via binding to NLRP3 protein, which is considered to be the target of itaconate and its derivative 4-octyl itaconate (4-OI) [77]. A study in 2018 showed that multiple NLRP3 activators caused the disassembly of the trans-Golgi network into a dispersed trans-Golgi network, which could be a platform to contribute to the assembly and activation of NLRP3 inflammasome [78]. Additionally, the expression and activity of NLRP3 inflammasome can be regulated by diverse mechanisms, for instance, PTMs, microRNAs, and endogenous modulators like CARD-only proteins and pyrin-only proteins [79,80,81]. From this perspective, regulatory mechanisms underlying inflammasome activation may be more intricate than prior appreciation, and they seem likely to vary according to the type of cell and stimulus [82]. Therefore, future research should further elucidate the molecular mechanisms involved in inflammasome.

Interestingly, inflammasome-mediated pyroptosis and inflammation appear to exhibit distinct manifestations at different stages of AAA progression. As a study indicated in 2019, the expression frequencies of NLRP3, AIM2, and caspase-5 in macrophages and lymphocytes were significantly lower in samples from the control group than from the AAA group. However, as AAA lesions progressed, the expression and activity of inflammasomes gradually decreased [83]. Part of the explanation may be that itaconate confers tolerance to late inflammasome activation. As shown by Bambouskova et al. [84], the accumulation of endogenous itaconate in response to prolonged lipopolysaccharide (LPS) stimulation blocked caspase-1 activation and GSDMD processing, but it was also found that the Cys77 of GSDMD might be the target of itaconate modification produced by mouse bone marrow-derived macrophages (BMDMs). Another study reported that necrotic cell debris from autologous cells promoted the expression of AIM2 and NLRP3 inflammasomes in the VSMCs of advanced AAA tissue, followed by activating downstream inflammatory flare, suggesting that inflammasome might serve as a “bridge” between VSMC dysfunction and inflammatory response [85].

## 7. Potential Drugs Targeting Inflammasome for the Treatment of AAA

The above mechanism studies demonstrate that inflammasome is so involved in the occurrence and development of AAA that it could become a potential target for drug treatment of AAA. Based on the complicated regulatory mechanisms of inflammasome activation, the upstream signaling, assembly of inflammasome, downstream caspase-1 activation, GSDMD cleavage, and inflammatory cytokines produced by inflammasome may all become the targets for the direct or indirect inhibition of inflammasome. In practice, the CANTOS trial using canakinumab revealed that IL-1-targeted therapy was effective in preventing atherothrombotic events [86]. Nevertheless, canakinumab is not only costly, but the long-term inhibition of IL-1 signaling also has the potential to cause adverse events, for instance, infection and immune homeostasis disorder [82]. Additionally, ASC and caspase-1 are involved not only in NLRP3 inflammasome but also in other inflammasomes that contribute to pathogen clearance (e.g., NLRP1 and AIM2 inflammasomes). Hence, pharmacological inhibitors that more specifically target the inflammasome could be a better choice to treat inflammasome-related diseases. Recently, several studies have reported the effectiveness of pharmacological inhibitors targeting NLRP3 inflammasome or AIM2 inflammasome in the treatment of experimental AAAs (Table 1).

### 7.1. MCC950 

MCC950 is currently the most well-studied potent, selective small-molecule NLRP3 inhibitor (also called CP-456,773 or CRID3) [35]. Surprisingly, MCC950 exhibits high selectivity towards NLRP3, which does not impede any of pathways driven by other inflammasomes. Moreover, it suppresses all known stimuli that can cause NLRP3 activation to date [98]. A previous study found that MCC950 could block pro-IL-1β processing mediated by caspase-1 [99]. Subsequently, Coll et al. [100] also demonstrated that MCC950 was capable of blocking both canonical and non-canonical pathways of NLRP3 inflammasome activation as well as IL-1β production by eliminating ASC oligomerization in mouse and human macrophages. Strikingly, a recent study pointed out that its mechanism is due to the direct binding of MCC950 to the Walker B motif on the NACHT domain of NLRP3, followed by blocking ATP hydrolysis and NLRP3 inflammasome formation [87]. Based on these, as Ren et al. [45] described, the use of MCC950 blocked the expression and activity of MMP-9 in macrophages, reduced the degradation of VSMCs’ contractile proteins and ECM damage, and ultimately inhibited the formation of aortic aneurysm in Ang II-infused hypercholesterolemic mice. Regrettably, the clinical development of MCC950 has been halted because of its hepatotoxicity [98].

### 7.2. Glyburide

Glyburide is a sulfonylurea-based compound used to treat type 2 diabetes (T2D). Its mechanism of action involves the inhibition of ATP-sensitive K^+^ (K^+^_ATP_) channels in pancreatic β cells to stimulate insulin secretion [101]. A previous study revealed that glyburide could prevent PAMPs, DAMPs, and the crystal-induced activation of NLRP3 inflammasome in BMDMs, while it did not alter NLRP3 expression or suppress AIM2 or NLRC4, suggesting that glyburide suppressed NLRP3 upstream of ASC [88]. Moreover, glyburide also effectively blocked caspase-1 activation and IL-1β secretion when tested against stimuli that are not dependent on the P2X7 receptor but require TLR4 signaling, indicating that it functions downstream of the P2X7 receptor [88]. In addition, as mentioned above, in 2017, Wu et al. [44] also observed that glyburide inhibited the degradation of VSMCs’ contractile proteins mediated by NLRP3 inflammasome-caspase-1, reducing aneurysmal aortic diameter and aneurysm incidence. Nonetheless, glyburide exerts its inhibitory effect at a considerably high in vivo dose, which can induce the occurrence of hypoglycemia, so its use remains limited to T2D [98].

### 7.3. Tranilast 

Tranilast, a tryptophan metabolite analogue, is also a specific inhibitor of NLRP3 inflammasome but has no effect on NLRC4 or AIM2 inflammasomes. Indeed, it has been revealed that tranilast blocked NLRP3 oligomerization via binding to the NLRP3 NACHT domain and abolishing the direct NLRP3–NLRP3 interaction so that it reduced caspase-1 activation and IL-1β secretion [89]. Moreover, tranilast impairs the endogenous NLRP3–ASC interaction but not the NLRP3-NEK7 interaction, thus increasing its likelihood of directly targeting NLRP3. Furthermore, it does not hinder the upstream signaling events of NLRP3 inflammasome, such as NLRP3 and pro-IL-1β expression, K^+^ efflux, chloride efflux, ROS production, and mitochondrial damage [90]. As early as 2008, a study found that the administration of tranilast could suppress the CaCl_2_-induced dilation of the AAA arterial wall, which is associated with the retention of medial elastin, the attenuation of mural mast cells and lymphocyte infiltration, reduction in neovascularization, and decreased MMP-9 activity [91]. Tranilast is a reasonably safe compound that patients have shown good tolerance of when tested at high dose levels [102]. Given its high safety profile in clinic, it may have important implications for treating NLRP3-driven diseases.

### 7.4. Bay 11-7082 

Bay 11-7082 is a phenyl vinyl sulfone that inhibits the NF-κB pathway by blocking the kinase activity of inhibitor of nuclear factor kappa B kinase subunit beta (IKKβ) [103]. As Ren et al. [92] reported, in a rat AAA model perfused with PPE, the addition of Bay 11-7082 silenced adipocyte enhancer-binding protein 1 (AEBP1) in VSMCs, resulting in NF-κB pathway inhibition and the reduced expression of inflammatory factors and MMPs, followed by the inhibition of the progression of AAA. Although this effect could theoretically suppress the priming stage of NLRP3 via inhibiting the activation of NF-κB, there is evidence that the inhibition of LPS-induced NLRP3 inflammasome activation in macrophages might not be related to this [93]. The mechanism may be explained by the fact that, as a Michael receptor, Bay 11-7082 alkylates cysteine residues in the ATPase domain of NLRP3, blocking NLRP3 ATPase activity, thus inhibiting NLRP3-induced ASC oligomerization. Moreover, when the vinyl sulfone moiety of Bay 11-7082 was removed, it changed from active to inactive. Therefore, compared with other inflammasomes, Bay 11-7082 also showed the selective inhibition of NLRP3 inflammasome [93]. Vinyl sulfone derivatives have been used as antiparasitic agents in dogs and mice over recent years [104], and these preclinical trials have suggested that this class of compounds is well tolerated, non-mutagenic, and has an appropriate pharmacokinetic profile as well as readily penetrating cell membranes [93]. Bay 11-7082 and other vinyl sulfone derivatives represent good candidates for the drug treatment of inflammasome-related diseases.

### 7.5. Andrographolide

Andrographolide is an active component extracted from *Andrographis paniculate*, which has multifarious pharmacological effects [105]. Previously, one study showed that andrographolide inhibited the activation of the NF-κB pathway in macrophages and VSMCs, thereby attenuating the infiltration of inflammatory cells in the aorta by down-regulating NF-κB-mediated cytokine production and α4 integrin expression, which ultimately suppressed the progression of the PPE-perfused AAA model in mice [94]. Andrographolide also regulated the ubiquitination type of target protein puromycin-sensitive aminopeptidase (PSAP) by binding to PSAP 577-919, which shifted PSAP from K63 ubiquitination modification that promoted TLR4-NF-κB signaling to K48 ubiquitination modification and oligomerization. In turn, PSAP interacted with the NOD-LRR domain of NLRP3 through 1-577 to block inflammasome assembly and thus inhibit caspase-1 activation, realizing a short-circuit regulation of the inflammatory response. In addition, the therapeutic effect of andrographolide may also be associated with the inhibition of AIM2 inflammasome. As reported by Gao et al. [95], andrographolide significantly impeded the activation of AIM2 inflammasome and pyroptosis in macrophages via preventing AIM2 translocation into the nucleus to sense radiation-induced dsDNA damage, thereby mitigating radiation-induced lung inflammation and fibrosis. Andrographolide has been used clinically for many years, with low toxicity and known pharmacokinetic parameters [105]. In conclusion, andrographolide provides a potential pharmacological opportunity for us to delay disease progression in AAA patients.

### 7.6. Quercetin

Quercetin is a flavonoid with anti-inflammatory activity [106]. In 2012, it was found that in the CaCl_2_-induced mouse AAA model, macrophage and CD3^+^ T cell infiltration in aortic tissues, the activation of NF-κB pathway, and inflammatory cytokine release were all inhibited after quercetin treatment [96]. Notably, quercetin reduced the expression of MMP-2, MMP-9, cathepsin B, and cathepsin K while promoting the tissue inhibitor of metalloproteinase (TIMP)-1 gene expression, subsequently hindering the expansion and progression of AAA [96]. Over recent years, several studies have indicated that quercetin could indirectly inhibit the expression and activation of NLRP3 inflammasome via inhibiting NF-κB pathway activation and scavenging ROS, thus playing a protective role in various inflammatory diseases [106,107,108]. In 2017, Domiciano et al. [97] further revealed that quercetin could also directly block the oligomerization of the inflammasome component ASC in macrophages, followed by inhibiting the assembly and activation of NLRP3 inflammasome and AIM2 inflammasome, which was evidenced by its inhibition of constitutive activated NLRP3 inflammasome. As a consequence, it reduced IL-1β secretion mediated by NLRP3 inflammasome and AIM2 inflammasome but not NLRC4 inflammasome in a dose-dependent manner, suggesting that the inhibition of activation signals by quercetin was limited to ASC-dependent inflammasomes [97]. In summary, these studies indicate that quercetin may block inflammasome activation in a variety of ways, which holds great promise for treating multiple inflammatory diseases such as AAA.

## 8. Conclusions

Inflammasome is the central node of immune sensing within the innate immune system, and aberrant inflammasome activation is the core aspect of deleterious chronic inflammation. Accumulating evidence suggests that inflammasomes such as NLRP3 inflammasome and AIM2 inflammasome contribute to the pathogenesis of AAA, a chronic inflammatory disease, and are considered promising targets to prevent or treat this disease. Therefore, the studies that profoundly explore the role of inflammasome in AAA can enhance our understanding of the relationship between them and hopefully guide our clinical drug treatment of AAA. Here, we also analyzed some pharmacological inhibitors targeting NLRP3 inflammasome or AIM2 inflammasome discovered in recent years and described their mechanisms of action and therapeutic potential for experimental AAAs. While these studies on AAA are mostly still at the drug discovery stage (candidate drug development), these candidates have great potential for future in-depth research and even the treatment of clinical AAA due to the lack of FDA (U.S. Food and Drug Administration)- or CFDA (China Food and Drug Administration)-approved drug therapies to limit the progression of AAA or reduce the risk of AAA rupture. In conclusion, we expect future studies to pay more attention to demonstrating comprehensively and explicitly how the mechanisms underlying inflammasome enhance the progression of AAA as well as explore the prospect of treating clinical AAA via pharmacological inhibitors targeting inflammasome so that certain potential ideal drugs can be developed and applied as soon as possible, although no selective inhibitor of inflammasome has been approved for clinical treatment at present.

## Figures and Tables

**Table 1:** Influence of pharmacological inhibitors targeting NLRP3 inflammasome or AIM2 inflammasome on experimental AAAs.

Drug Name	Animal Species and Strain	AAA Model	Potential Mechanism	Experimental Effect	Cite
MCC950	Wild-type C57BL/6 mice	Ang II infusion and high-fat diet	Block the ATPase domain of NLRP3 Suppress both canonical and non-canonical pathways of NLRP3 inflammasome activation	Blocked the expression and activity of MMP-9 in macrophages Reduced the degradation of VSMCs’ contractile proteins and ECM damage Inhibited the formation of AAA	[45] [87]
Glyburide	Wild-type C57BL/6 mice	Ang II infusion and high-fat diet	Inhibit ATP-sensitive K^+^ channels Suppress ASC aggregation downstream of P2X7 receptor	Inhibited the degradation of VSMCs’ contractile proteins mediated by NLRP3 inflammasome-caspase-1 Reduced aneurysmal aortic diameter and aneurysm incidence	[44] [88]
Tranilast	Wild-type Sprague Dawley rats	CaCl_2_ induction	Bind to NLRP3 NACHT domain Abolish the direct NLRP3-NLRP3 interaction Impair the endogenous NLRP3-ASC interaction	Attenuated aortic infiltration of mast cells and T cells Decreased MMP-9 activity Reduced aneurysmal aortic dilation and preserved medial elastin	[89] [90] [91]
Bay 11-7082	Wild-type Sprague Dawley rats	PPE perfusion	Block kinase activity of IKKβ to inhibit NF-κB pathway Alkylate cysteine residues in the ATPase domain of NLRP3 Inhibit NLRP3 ATPase activity	Silenced AEBP1 in VSMCs Reduced the expression of inflammatory factors and MMPs Inhibited the progression of AAA	[92] [93]
Andrographolide	Wild-type C57BL/6 mice	PPE perfusion	Regulate PSAP ubiquitination type by binding to PSAP 577-919 Inhibit the activation of the NF-κB pathway and block NLRP3 inflammasome assembly Prevent AIM2 translocation into the nucleus to sense damaged dsDNA	Down-regulated NF-κB-mediated cytokine production and α4 integrin expression Attenuated the infiltration of inflammatory cells in the aorta Suppressed the progression of AAA	[94] [95]
Quercetin	Wild-type C57BL/6 mice	CaCl_2_ induction	Inhibit the activation of NF-κB pathway and scavenge ROS Block the oligomerization of ASC to inhibit the assembly and activation of NLRP3 inflammasome and AIM2 inflammasome	Inhibited inflammatory cell infiltration and cytokine release Reduced the expression of MMPs and cathepsins while promoting TIMP-1 gene expression Hindered the expansion and progression of AAA	[96] [97]

AAA, abdominal aortic aneurysm; AEBP1, adipocyte enhancer-binding protein 1; AIM2, absent in melanoma 2; Ang II, angiotensin II; ASC, apoptosis-associated speck-like protein containing a caspase recruitment domain; CaCl_2_, calcium chloride; dsDNA, double-stranded DNA; ECM, extracellular matrix; IKKβ, inhibitor of nuclear factor kappa B kinase subunit beta; MMPs, matrix metalloproteinases; NACHT, nucleotide-binding oligomerization domain; NF-κB, nuclear factor kappa-light-chain-enhancer of activated B cells; NLRP3, nucleotide-binding oligomerization domain-like receptor family pyrin domain-containing 3; PPE, porcine pancreatic elastase; PSAP, puromycin-sensitive aminopeptidase; ROS, reactive oxygen species; TIMP, tissue inhibitor of metalloproteinase; VSMCs, vascular smooth muscle cells.

## Data Availability

Not applicable.
